# Comparison of *Streptococcus pneumoniae* isolates occurring in optochin-susceptible and optochin-resistant variants by analyzing whole-genome sequencing data

**DOI:** 10.1128/spectrum.01939-24

**Published:** 2025-02-25

**Authors:** Sandra Vohrnová, Jana Kozáková, Michal Honskus

**Affiliations:** 1 The National Reference Laboratory for Streptococcal Infections, Department of Air-Borne Bacterial Infections, Centre for Epidemiology and Microbiology, National Institute of Public Health, Prague, Czech Republic; 2 Third Faculty of Medicine, Charles University, Prague, Czech Republic; 3 The National Reference Laboratory for Meningococcal Infections, Department of Air-Borne Bacterial Infections, Centre for Epidemiology and Microbiology, National Institute of Public Health, Prague, Czech Republic; Michigan State University, East Lansing, Michigan, USA

**Keywords:** *Streptococcus pneumoniae*, optochin, whole genome sequencing

## Abstract

The paper presents the study of a set of isolates of *Streptococcus pneumoniae*, which comprised two heterogeneous subpopulations, one of which was susceptible and the other resistant to optochin. The aim of the study was to compare the results of serotyping, multilocus sequence typing (MLST), ribosomal multilocus sequence typing (rMLST), and variation analysis of these subpopulations and to investigate the genetic probable causes of optochin resistance. The strains studied were cultured from samples taken from patients with invasive pneumococcal disease in the Czech Republic in 2019 and 2020. A total of 10 studied pairs of isolates were subject to serotyping and whole-genome sequencing (WGS). None of the typing methods (serotyping, MLST, or rMLST) applied to pairs of optochin-susceptible and optochin-resistant isolates revealed differences in serotype, sequence type, or ribosomal sequence type. The WGS data analysis identified point mutations in ATP (adenosine triphosphate) synthase genes in 8 of the 10 optochin-resistant isolates. In seven optochin-resistant isolates, the mutation was found in the *atp*C gene and in one isolate in the *atp*A gene. One of the mutations in the *atp*C gene has not yet been published in the literature; it is a mutation at position 143T > C with an amino acid change of Val48Ala. In 8 out of the 10 optochin-resistant isolates, the possible genetic basis for resistance was identified, involving point mutations in the *atp*A and *atp*C genes. In the remaining two isolates, no clear genetic explanation for the optochin resistance in *S. pneumoniae* was found, based on current knowledge.

**IMPORTANCE:**

Globally, among the most fundamental tests used for the identification of *Streptococcus pneumoniae* isolates is determining susceptibility to optochin. In the last 2 decades, optochin-resistant strains have been frequently reported in the literature, which can lead to the misidentification of *S. pneumoniae*. This study compares whole-genome sequencing data of optochin-susceptible and optochin-resistant subpopulations of *S. pneumoniae* isolates and investigates the genetic probable causes of resistance in the genomes of optochin-resistant subpopulations.

## INTRODUCTION

*Streptococcus pneumoniae* (pneumococcus) is a gram-positive, encapsulated, catalase-negative, facultative anaerobic coccus often occurring in pairs or in chains. On the blood agar, it creates alpha-hemolysis. The basic identification of *S. pneumoniae* is based on its bile solubility (10% sodium deoxycholate solution), its sensitivity to optochin, and molecular-genetic methods such as testing for the presence of the capsular polysaccharide (*cpsA*) gene, the autolysine (*lytA*) gene, and pneumolysine (*ply*) genes (1, 2).

Optochin, or ethylhydrocupreine hydrochloride, is a quinine derivative that acts on ATP (adenosine triphosphate) synthase bound to the membrane of a bacterium. ATP synthase is a transmembrane protein complex responsible for ATP synthesis in the presence of an ion gradient. ATP synthase is composed of two domains (F0 and F1), which are made up of several subunits. When hydrogen ions pass through the ATP synthase channel, the rotating mechanism of ATP synthase (the so-called “molecular machine”) is spun, revealing the reaction sites that allow ATP synthesis (3). Optochin blocks the described process by binding to ATP synthase structures and thus inhibits ATP synthesis in the cell (4, 5).

The investigation of optochin as a possible chemotherapy agent or possible active substance in the *S. pneumoniae* identification test has had a long history. In 1911, a study describing the effect of the use of optochin in the treatment of pneumococcal disease was published (6), but due to serious adverse effects, its use for the treatment of patients was abandoned (7). In 1915, the action of optochin on strains of *S. pneumoniae* and other strains of the genus *Streptococcus*, specifically viridans streptococci, was introduced. *S. pneumoniae* proved to be susceptible to optochin, unlike other viridans streptococci, which are resistant to optochin (8). This knowledge was applied in the 1950s, when optochin began to serve as the active substance in one of the main identification tests of *S. pneumoniae* (9–11). In the 1980s, the first cases of *S. pneumoniae* resistance to optochin were described (12, 13). Subsequently, mutations in the *atpA* (ATP synthase, a-subunit) gene, which encodes the a-subunit of ATP synthase, and in the *atp*C (ATP synthase, c-subunit) gene, which encodes the c-subunit of ATP synthase, were found to be responsible for *S. pneumoniae* resistance to optochin (4).

This study presents an analysis of a set of 10 *S*. *pneumoniae* isolates from invasive pneumococcal diseases delivered to the National Reference Laboratory for Streptococcal Infections (NRL/STR) in 2019 and 2020, in which a dual cell subpopulation was found when cultured on blood agar—part of the cells were optochin-susceptible (OptS) and part optochin-resistant (OptR). The subpopulation of optochin-resistant *S. pneumoniae* grew as single colonies in the inhibition zone around the optochin disk. From each such isolate, one colony from the inhibition zone was subcultured onto a new agar plate, and the optochin susceptibility test was repeated. It was confirmed that within a single isolate, two subpopulations were present—one susceptible to optochin and the other resistant to optochin. The isolates were subsequently serotyped, and then whole-genome sequencing (WGS) was performed to compare optochin-susceptible and optochin-resistant isolates and to determine the genetic probable cause of the resistance.

## MATERIALS AND METHODS

### Isolates of *Streptococcus pneumoniae*

The study included 10 isolates of *S. pneumoniae* obtained from patients with invasive pneumococcal disease—each of the isolates studied included an optochin-susceptible and optochin-resistant subpopulation of cells. Isolates were delivered to the NRL/STR within the framework of the Invasive Pneumococcal Disease (IPD) surveillance programme in the Czech Republic. Of all the isolates obtained for the IPD surveillance programme in 2019 and 2020, only those in the study exhibited dual sensitivity to optochin. Table 1 summarizes information on the sex and age of the patient with IPD and year of isolation of the strains. The isolates were collected from various cities throughout the Czech Republic.

**TABLE 1:** Summary characteristics of the set of the studied isolates, Czech Republic, 2019 and 2020^*a*^

Isolate	NCBI accession	PubMLST ID	Gender	Age group (years)	Region	Material	Disease	Solubility in bile	Susceptibility to optochin	Presence of *cpsA* gene	Serotype	ST^*b*^	rST^*c*^
672/2019C	SAMN45030567	158059	Male	40–64	CZ064	Blood	Bacteremia	+	Susceptible	+	12F	15340	12327
672/2019R	SAMN45030568	158060	Male	40–64	CZ064	Blood	Bacteremia	+	Resistant	+	12F	15340	12327
721/2019C	SAMN45030569	158061	Female	40–64	CZ080	Autopsy material–brain smear	Sepsis	+	Susceptible	+	22F	433	13404
721/2019R	SAMN45030570	158062	Female	40–64	CZ080	Autopsy material–brain smear	Sepsis	+	Resistant	+	22F	433	13404
729/2019C	SAMN45030571	158063	Female	65+	CZ072	Blood	Pneumonia	+	Susceptible	+	10A	1551	12356
729/2019R	SAMN45030572	158064	Female	65+	CZ072	Blood	Pneumonia	+	Resistant	+	10A	1551	12356
763/2019C	SAMN45030573	158065	Male	1–4	CZ031	Pus from the mastoid antrum	Otoantritis	+	Susceptible	+	23A	992	12435
763/2019R	SAMN45030574	158066	Male	1–4	CZ031	Pus from the mastoid antrum	Otoantritis	+	Resistant	+	23A	992	12435
95/2020C	SAMN45030575	158067	Female	65+	CZ080	Blood	Pneumonia	+	Susceptible	+	19A	416	11332
95/2020R	SAMN45030576	158068	Female	65+	CZ080	Blood	Pneumonia	+	Resistant	+	19A	416	11332
196/2020C	SAMN45030577	158069	Female	65+	CZ020	Blood	Pneumonia	+	Susceptible	+	9N	66	178094
196/2020R	SAMN45030578	158070	Female	65+	CZ020	Blood	Pneumonia	+	Resistant	+	9N	66	178094
322/2020C	SAMN45030579	158071	Male	40–64	CZ072	Blood	Sepsis	+	Susceptible	+	19A	994	178093
322/2020R	SAMN45030580	158072	Male	40–64	CZ072	Blood	Sepsis	+	Resistant	+	19A	994	178093
358/2020C	SAMN45030581	158073	Male	40–64	CZ080	Blood	Pneumonia	+	Susceptible	+	9N	66	12230
358/2020R	SAMN45030582	158074	Male	40–64	CZ080	Blood	Pneumonia	+	Resistant	+	9N	66	12230
359/2020C	SAMN45030583	158075	Female	40–64	CZ064	Blood	Pneumonia	+	Susceptible	+	4	2190	24928
359/2020R	SAMN45030584	158076	Female	40–64	CZ064	Blood	Pneumonia	+	Resistant	+	4	2190	24928
363/2020C	SAMN45030585	158077	Male	65+	CZ010	Blood	Bacteremia	+	Susceptible	+	11A	14712	644
363/2020R	SAMN45030586	158078	Male	65+	CZ010	Blood	Bacteremia	+	Resistant	+	11A	14712	644

^*a*^ C, susceptible; R, resistant; +, positive.

^*b*^ Sequence type.

^*c*^ Ribosomal sequence type.

*S. pneumoniae* isolates from patients with IPD were sent to the NRL/STR by clinical laboratories in compliance with Czech legislation.

### Identification of *Streptococcus pneumoniae*

Ten pairs of OptS/OptR subpopulations were cultured overnight on the blood agar (Columbia agar with sheep blood, Oxoid) at 35°C in a 5% CO_2_ atmosphere with a diagnostic optochin disk applied (Optochin 5 µg [OP5] Diagnostic Discs, Oxoid). Subsequently, the presence of an inhibition zone was evaluated. Optochin-susceptible isolates exhibited an inhibition zone of 14 mm or more in diameter; no inhibition zone was observed in optochin-resistant isolates. For confirmation, the optochin susceptibility test was repeated twice. Confirmation of the identification of *S. pneumoniae* was assessed by the presence of alpha-hemolysis on blood agar, and bile solubility test (BD BBL Desoxycholate Reagent Droppers, Becton, Dickinson and Company).

For long-term preservation, strains were cultured overnight on the blood agar (Columbia agar with sheep blood, Oxoid) at 35°C in a 5% CO_2_ atmosphere before harvesting and then stored in cryo-chambers with glycerol broth (ITEST KRYOBANKA B, ITEST plus) at −70°C in the NRL/STR collection.

### Genomic DNA isolation and detection of capsular types

Genome DNA of the samples studied was isolated using the QIAamp DNA Mini Kit (QIAGEN) with a modified protocol for gram-positive bacteria. Serotyping of the isolates was performed by a combination of quellung reaction and end-point multiplex polymerase chain reaction (mPCR) according to the published procedure (14). The mPCR performed included primers for the capsular polysaccharide gene *cps*A, the presence of which provided further confirmation of the correct identification of *S. pneumoniae*.

### WGS and WGS data processing

WGS was performed at the European Molecular Biology Laboratory (Heidelberg, Germany) using the whole-genome sequencer Illumina MiSeq. The WGS data were processed using the Velvet *de novo* Assembler software with optimization performed by the script Velvet-Optimiser (15). The assembled genomes of the isolates were uploaded to the PubMLST international database (https://pubmlst.org/organisms/streptococcus-pneumoniae), which uses the BIGSdb (Bacterial Isolate Genome Sequence Database) platform (16). Multilocus sequence typing (MLST) alleles, sequence type (ST), ribosomal MLST (rMLST) alleles, and rST were automatically determined by PubMLST database (17–20). Further processing of WGS data was performed using the online bioinformatics platform Bacterial and Viral Bioinformatics Resource Center (https://www.bv-brc.org/) (21). The Comprehensive Genome Analysis tool was employed to identify the genes present in the bacterial genome. Then, the variation analysis tool was used with a predefined setting applied, using the Burrows-Wheeler Alignment Tool (BWA-MEM) and the SNP caller Freebayes (SNP [single nucleotide polymorphism], point mutation) (22, 23). The quality of the mutations was predefined in the program with a read depth greater than 5 and an SNP quality greater than 10 (QUAL). The results obtained were further manually checked, and the high quality of the mutations detected was guaranteed by more stringent mutation quality parameters—results with a read depth less than 30 and a variant fraction less than 0.6 were excluded. The optochin-susceptible isolate from each pair of OptS and OptR isolates always served as the reference isolate.

## RESULTS

### Identification and serotyping results

All the samples of both OptS and OptR isolates were identified as *S. pneumoniae*, produced alpha-hemolysis on the blood agar, were bile soluble, and contained the *cpsA* gene. The isolates differed within pairs only in response to optochin, with OptS isolates showing a zone of inhibition of 14 mm or more on the blood agar and OptR isolates showing no zone of inhibition on agar. Within the pairs, OptS and OptR isolates had the same serotype (Table 1). Serotypes 9N and 19A occurred twice out of the 10 pairs of isolates, with the other serotypes recorded only once (4, 10A, 11A, 12F, 22F, and 23A).

### WGS results

The parameters of the whole-genome sequencing are presented in Table 2. The average genome size was 2.1 Mbp, the lowest number of contigs being 43, and the highest number reaching 112.

**TABLE 2:** Details of whole-genome sequencing

Isolate	Number of contigs	Total length	Maximum length	Mean length	N50
672/2019C	50	2,004,027	383,489	40,081	94,336
672/2019R	54	2,003,624	383,426	37,105	84,953
721/2019C	54	2,043,738	194,068	37,847	77,722
721/2019R	104	1,949,887	88,797	18,749	35,340
729/2019C	71	2,030,947	150,065	28,605	58,246
729/2019R	51	2,058,075	198,423	40,355	109,114
763/2019C	43	2,116,103	347,953	49,212	130,143
763/2019R	47	2,116,222	436,536	45,026	188,643
95/2020C	53	2,111,463	418,468	39,839	227,220
95/2020R	65	2,097,246	289,748	32,266	84,944
196/2020C	51	2,058,116	341,288	40,356	125,600
196/2020R	52	2,057,932	390,953	39,576	125,593
322/2020C	87	2,169,719	182,169	24,940	70,540
322/2020R	108	2,132,928	171,249	19,750	48,628
358/2020C	54	2,067,986	261,377	38,297	122,435
358/2020R	61	2,058,155	217,516	33,741	73,041
359/2020C	112	2,041,542	105,199	18,229	47,988
359/2020R	70	2,053,382	149,556	29,335	64,949
363/2020C	43	2,002,967	279,634	46,581	120,153
363/2020R	54	1,994,386	279,399	36,934	85,898

In every pair of OptS and OptR isolates, the MLST and rMLST results were consistently identical (Table 1). In the 10 pairs of OptS and OptR, nine different sequence types were identified (ST-66, ST-416, ST-433, ST-992, ST-994, ST-1551, ST-2190, ST-14712, and ST-15340). Two pairs of isolates of serotype 9N had the same ST-66 but different rST, while two pairs of isolates of serotype 19A had different ST and rST.

Further analysis was focused on comparing pairs of “tested” genomes of OptR isolates with the “reference” genomes of OptS isolates. Variation analysis revealed that the compared sequences varied in the number of mutations present—the difference between the genomes of OptS and OptR pairs of isolates was at least 23 mutations and the highest one reached 92 mutations, both synonymous, non-synonymous, and nonsense mutations. When additional, more stringent mutation quality parameters were applied and when mutations in genes for mobile element proteins and for hypothetical proteins were excluded, there were 0 (isolate 721/2019R) to 7 (isolate 363/2020R) non-synonymous variants in genes encoding proteins with known or presumed known function (Table 3). The analysis did not reveal any gene exhibiting mutations in all 10 OptR isolates. The most frequently found mutations were SNPs in genes coding for ATP synthase; it was present in 8 of the 10 OptR isolates. In seven OptR isolates, a mutation in the *atp*C gene was found, and in one case, a mutation was identified in the *atp*A gene. In our study, all point mutations found in ATP synthase genes but one have already been described. To the best of our knowledge, this is the first study reporting 143T > C substitution in *atp*C gene (Val48Ala) (Table 4).

**TABLE 3:** Detected non-synonymous mutations in genes for proteins of known and putative known function^*a*^

Isolate	Gene ID	Gene product	Mutation identified	Change in amino acids
672/2019R	fig|1313.43129.peg.453	Phosphate ABC transporter, ATP-binding protein PstB (TC 3.A.1.7.1)	541T > C	Ser181Pro
672/2019R	fig|1313.43129.peg.1662	KtrAB potassium uptake system, peripheral membrane component KtrA	310C > T	Gln104^*b*^
721/2019R	–	–		
729/2019R	fig|1313.43133.peg.210	ATP synthase F0 sector subunit c (EC 3.6.3.14)	145G > T	Ala49Ser
763/2019R	fig|1313.43134.peg.478	ATP synthase F0 sector subunit c (EC 3.6.3.14)	143T > C	Val48Ala
763/2019R	fig|1313.43134.peg.1431	Pyrrolidone-carboxylate peptidase (EC 3.4.19.3)	148G > T	Ala50Ser
763/2019R	fig|1313.43134.peg.2146	Cell wall surface anchor family protein	39G > C	Lys13Asn
95/2020R	fig|1313.43137.peg.1380	Translation initiation factor 3	217G > C	Ala73Pro
95/2020R	fig|1313.43137.peg.2147	ATP synthase F0 sector subunit c (EC 3.6.3.14)	145G > T	Ala49Ser
196/2020R	fig|1313.43120.peg.674	ATP synthase F0 sector subunit c (EC 3.6.3.14)	69G > T	Met23Ile
196/2020R	fig|1313.43120.peg.1643	Glycosyl hydrolase family 29 (alpha-L-fucosidase)	1517C > T	Ala506Val
322/2020R	fig|1313.43121.peg.124	PTS system, galactose-specific IIA component (EC 2.7.1.204)	466G > A	Ala156Thr
322/2020R	fig|1313.43121.peg.2076	ATP synthase F0 sector subunit c (EC 3.6.3.14)	142G > T	Val48Phe
322/2020R	fig|1313.43121.peg.2200	N-terminal acetyltransferase-related protein	302C > T	Ala101Val
358/2020R	fig|1313.43119.peg.8	ATP synthase F0 sector subunit c (EC 3.6.3.14)	145G > A	Ala49Thr
358/2020R	fig|1313.43119.peg.2127	Predicted cell wall-anchored protein SasA (LPXTG motif)	1269GGCT > AGTC	SerAla423SerVal
359/2020R	fig|1313.43124.peg.148	ATP synthase F0 sector subunit c (EC 3.6.3.14)	142G > T	Val48Phe
359/2020R	fig|1313.43124.peg.60	Uncharacterized protease YrrO	458T > C	Val153Ala
359/2020R	fig|1313.43124.peg.1241	Putative membrane-spanning protein	322A > G	Asn108Asp
359/2020R	fig|1313.43124.peg.1885	Cell wall surface anchor family protein	1165T > G	Leu389Val
359/2020R	fig|1313.43124.peg.1885	Cell wall surface anchor family protein	2463CGAC > TGAG	GlyAsp821GlyGlu
363/2020R	fig|1313.43126.peg.626	Uncharacterized MFS-type transporter	832A > G	Thr278Ala
363/2020R	fig|1313.43126.peg.892	ABC transporter, permease protein (cluster 3, basic aa/glutamine/opines)	528A > G	Ile176Met
363/2020R	fig|1313.43126.peg.986	Manganese ABC transporter, inner membrane permease protein SitD	225C > G	Phe75Leu
363/2020R	fig|1313.43126.peg.1549	Pyrrolidone-carboxylate peptidase (EC 3.4.19.3)	199T > G	Trp67Gly
363/2020R	fig|1313.43126.peg.1562	Maltodextrin ABC transporter, permease protein MdxG	560A > G	Ter187Trpext*^c^?
363/2020R	fig|1313.43126.peg.1631	Tellurite methyltransferase (EC 2.1.1.265)	753A > G	Ter251Trpext*^c^?
363/2020R	fig|1313.43126.peg.1941	ATP synthase F0 sector subunit a (EC 3.6.3.14)	618G > T	Trp206Cys

^*a*^ R, resistant; –, not availible.

^*b*^ Termination codon.

^*c*^ Mutation of the termination codon—addition of a new amino acid.

**TABLE 4:** Previously published mutations in ATP synthase genes and a newly reported mutation published in this study

Gene	Mutation	Number	Country
atpC	Met13→Val/Ile	2	Brazil, Tunisia [30Pinto, 32Raddaoui]
atpC	Gly14→Ser	2	Brazil, France [25Cogné, 30Pinto]
atpC	Val15→Ile	1	Tunisia [32Raddaoui]
atpC	Gly18→Ser	2	Brazil [30Pinto]
atpC	Gly20→Ser/Ala	4	Brazil, USA [26Pikis, 30Pinto]
atpC	Met23→Ile	5	Czech Republic (this study), Brazil, USA (24–26)
atpC	Ala31→Val	1	Brazil (26)
atpC	Met44→Ile	1	Japan (27)
atpC	Phe45→Leu/Val	3	Brazil (25, 26)
atpC	Gly47→Val	1	Argentina (28)
atpC	Val48→Ile/Phe	6	Czech Republic (this study), Tunisia, Japan (27, 29)
atpC	**Val48→Ala^*a*^**	1	Czech Republic (this study)
atpC	Ala49→Ser/Thr/Gly	24	Czech Republic (this study), Argentina, Brazil, Japan, USA, Russia (24–28, 30)
atpC	Phe50→Leu	1	Japan (27)
atpA	Met23→Val	1	Tunisia (31)
atpA	Val52→Ile	1	Tunisia (31)
atpA	Leu99→Ile	1	Tunisia (31)
atpA	Trp206→Cys/Ser	5	Czech Republic (this study), Brazil, USA, Argentina (24, 28, 32)

^*a*^ Highlighted, unpublished mutation.

In two OptR isolates, no mutation in ATP synthase genes was present. In isolate 672/2019R, a mutation in the gene for the phosphate ABC transporter ATP binding protein PstB and a mutation in the gene for the KtrAB potassium uptake system were found. When stringent mutation quality parameters were used, no mutations were detected in the genes encoding proteins with known or presumed functions in the isolate 721/2019R. When less stringent parameters were used, a mutation in the *comX* (competence-specific sigma factor ComX) gene was found.

In the study, mutations in non-coding regions of genomes (so-called intergenic regions) were also analyzed (Table 5). In isolate 672, mutations in the intergenic region upstream of the genes *mltG* (Murein endolytic transglycosylase) and *LivF* (branched-chain amino acid [BCAA] transport ATP-binding protein LivF) were detected. Isolate 721/2019R exhibited a mutation in an intergenic region upstream of the heterodimeric efflux ABC transporter.

**TABLE 5:** Detected mutations in intergenic regions upstream of the genes for proteins of known and putative known function^*a*^

Isolate	Position in the contig	Reference nucleotide	Variant nucleotide	“Upstream” gene	“Downstream” gene	Effect of the mutation
672/2019R	57292	A	C	Transcription elongation factor GreA	Murein endolytic transglycosylase MltG	Modifier
672/2019R	119	T	A		Branched-chain amino acid ABC transporter, ATP-binding protein LivF (TC 3.A.1.4.1)	Modifier
721/2019R	6846	T	C	Degenerate transposase	Heterodimeric efflux ABC transporter, multidrug resistance	Modifier
358/2020R	7	G	T		Predicted cell-wall-anchored protein SasA (LPXTG motif)	Modifier
359/2020R	49843	A	G	Hypothetical protein	Ribose-phosphate pyrophosphokinase (EC 2.7.6.1)	Modifier
359/2020R	49855	G	A	Hypothetical protein	Ribose-phosphate pyrophosphokinase (EC 2.7.6.1)	Modifier
359/2020R	49869	A	G	Hypothetical protein	Ribose-phosphate pyrophosphokinase (EC 2.7.6.1)	Modifier
363/2020R	1774	A	G	Aquaporin (major intrinsic protein family)	Cell wall surface anchor family protein	Modifier

^*a*^ R, resistant.

## DISCUSSION

Since the 1980s, isolates of optochin-resistant *S. pneumoniae* have been reported in the literature. Since the discovery that mutations in ATP synthase genes are behind resistance of *S. pneumoniae* to optochin, a number of mutations in ATP synthase genes have been published. The most frequently reported mutations were in the *atpC* gene (4, 24–32); publications reporting mutations in the *atpA* gene (28, 31, 33, 34) were less frequent.

Two patterns of optochin-resistant *S. pneumoniae* occurrence are described in the literature: either as a single population composed of OptR cells or as a mixed population consisting of subpopulations of OptS and OptR cells, which were detected in our study. Previous studies have described mutations in the genes coding for ATP synthase as the only difference between the OptS and OptR subpopulations, and these mutations were related to the OptR phenotype.

There are no known interactions between the subpopulations or how their coexistence is maintained. Acquiring resistance to optochin does not confer a significant existential advantage for *S. pneumoniae* (33). Since optochin is a chemotherapeutic agent not used for therapeutic purposes in human medicine, the emergence of a new mutation and an optochin-resistant subpopulation of *S. pneumoniae* within a single Petri dish may provide an immediate advantage for this subpopulation. However, there is no evidence that these strains have any chance of spreading further under laboratory conditions.

The causes of mutations in ATP synthase genes are not clearly established. *In vitro* experiments have shown that exposure of *S. pneumoniae* to subinhibitory penicillin concentrations increases the frequency of mutations leading to optochin resistance (35). Penicillin antibiotics are commonly used in the treatment of pneumococcal diseases. The effect of antibiotic therapy on the occurrence of optochin-resistant *S. pneumoniae* could be a subject of further research.

In this study, we examined the difference between optochin-susceptible and optochin-resistant subpopulations using serotyping and WGS data analysis. The isolates analyzed belonged to eight capsular types (Table 1). The serotypes of the isolates in this study are consistent with those commonly found in the Czech Republic (36), which supports the hypothesis that the distribution of serotypes among OptR *S. pneumoniae* strains is predominantly influenced by the epidemiological profile of the geographical area (27, 31, 33). In 10 pairs of OptS and OptR, 9 different STs and 10 different rSTs were determined. Two pairs of serotype 9N had the same ST but differed in rST, two pairs of serotype 19A differed in ST and rST. No association between optochin resistance and serotype, ST, or rST was identified in our data set, which is in line with previous knowledge (33).

When investigating potential genetic causes of optochin resistance in OptR isolates using their WGS data, multiple mutations were identified, including a mutation in the *atp*C gene in seven OptR isolates and a mutation in the *atp*A gene in one isolate. Based on existing literature, the mutations identified in the genes encoding ATP synthase may represent a genetic basis for optochin resistance. However, this study did not establish a causal relationship, which could be explored in future research.

In two OptR isolates, no mutations that are according to current knowledge associated with the optochin-resistance phenotype have been found. In isolate 672/2019R, a mutation in the gene for the phosphate ABC transporter ATP-binding protein PstB (ATP-binding cassette) that actively transports phosphate through the bacterial membrane and a mutation in the gene for the KtrAB potassium uptake system (K+ transporter) were found. Neither of these mutations has been linked to optochin resistance in *S. pneumoniae* in the published literature. In isolate 721/2019R, when using less stringent mutation quality parameters, a mutation in the *comX* gene affecting transcription was found. The *comX* gene is not associated with optochin resistance according to current knowledge. The emergence of optochin resistance in these two isolates may be related to mutations found in genes that encode proteins that do not yet have a defined function. Another possible explanation for optochin resistance is the presence of mutations in non-coding regions or in gene regulators, which may lead to a modification of gene transcription. Isolate 672/2019R exhibited mutation upstream from the gene *MltG*, which plays a role in peptidoglycan synthesis (37). MltG functions as a peptidoglycan terminase and is required for maintaining the normal distribution of glycan strand lengths; mutations in the *mltG* gene result in elongated glycans (37). In the case of isolate 672/2019R, a mutation upstream of the *mltG* gene could alter its function, leading to the production of elongated or otherwise altered glycan molecules in the peptidoglycan layer. The effect of altered peptidoglycan on the ATP synthase molecule is unknown, no connection with *S. pneumoniae* resistance to optochin was reported in the literature. Further investigation may be warranted (38). Other mutation in isolate 672/2019R was detected upstream of gene for ABC transporter LivF. The ABC transporter LivF mediates the uptake of BCAAs such as leucine, isoleucine, and valine; it does not function as an efflux pump (39). The BCAAs transporter plays a role in bacterial survival within the host organism, contributing to the overall fitness of the bacterium and possibly influencing pathogenesis and virulence (40, 41). A mutation upstream of the gene for LivF could result in the upregulation of its expression, thereby enhancing the ability of the bacterium to survive in environments containing antibiotics. In isolate 721/2019R, a mutation in the non-coding region that precedes the gene for heterodimeric efflux ABC transporter (ATP binding cassette transporter) was detected. This transporter is associated with bacterial multiresistance to antibiotics through the efflux of antibiotics and other foreign substances from the cell (42). The mutation found in the region upstream of this gene may have resulted in an increase in its transcription.

When evaluating point mutations and comparing genomes, it is necessary to consider the principle and quality of the WGS method itself. One possible influence on the output WGS data is the quality of each individual sequence run. It is further stated that sequencing by synthesis on the Illumina MiSeq device introduces an error of 0.1% into the nucleotide sequencing, which means that 99.9% of nucleotides in the sequence are determined correctly (43).

In a previously published study, a difference was found between the OptS and OptR isolates in the presence of the genes for maltose O-acetyltransferase (EC 2.3.1.79) and uridine phosphorylase (EC 2.4.2.3), where these genes were present in the OptS isolates but were not found in the OptR isolate (26). Our analysis found there was no difference between OptS and OptR pairs of isolates. Uridine phosphorylase was present in all isolates studied regardless of their susceptibility to optochin. Maltose O-acetyltransferase was found in five pairs of OptS and OptR isolates, while it was absent in five other pairs of OptS and OptR isolates. According to our findings, we cannot confirm the hypothesis that the genomes of OptS and OptR isolates differ by these two genes.

It was repeatedly reported in the literature that optochin resistance may occur in *S. pneumoniae* isolates (44). It is important to monitor these OptR strains as these can have important clinical implications. Laboratories performing the identification of *S. pneumoniae* should use, in addition to optochin susceptibility testing, other tests confirming the identification of *S. pneumoniae* (44, 45).

### Conclusions

Comparison of the whole-genome data from 10 pairs of optochin-susceptible and optochin-resistant isolates revealed that a mutation in the ATP synthase gene, which may underlie the resistance to optochin, was detected in 8 out of 10 optochin-resistant isolates. Mutations in the *atp*C gene were found in seven OptR isolates, while a mutation in the *atp*A gene was detected in one OptR isolate. A mutation in the *atp*C gene at the 143T > C position has not yet been reported in the literature. Another possible cause of resistance to optochin may lie in mutations in the intergenic regions, which could affect the expression of downstream genes; these causes may be the focus of further research. By comparing these findings with the results of serotyping, MLST, and rMLST, it can be inferred that the initial population was a single bacterial strain in which subsequently point mutations occurred, including mutations in the ATP synthase genes, with these mutations leading to a phenotypic change and the emergence of a subpopulation resistant to optochin. The cause of these molecular changes responsible for the phenotypic alterations remains unexplained and may be subject to further investigation.

## Data Availability

The raw data for the samples can be accessed in NCBI under BioProject accession number PRJNA1190237 and BioSample accessions SAMN45030567, SAMN45030568, SAMN45030569, SAMN45030570, SAMN45030571, SAMN45030572, SAMN45030573, SAMN45030574, SAMN45030575, SAMN45030576, SAMN45030577, SAMN45030578, SAMN45030579, SAMN45030580, SAMN45030581, SAMN45030582, SAMN45030583, SAMN45030584, SAMN45030585, SAMN45030586. Table 1 lists the BioSample accessions of the samples. The assembled genomes are available in the PubMLST database under the corresponding PubMLST ID numbers, as shown in Table 1.
